# The Outcome of Induction Chemotherapy, Followed by Neoadjuvant Chemoradiotherapy and Surgery, in Locally Advanced Rectal Cancer

**DOI:** 10.30699/IJP.2021.130482.2441

**Published:** 2021-05-09

**Authors:** Ali Yaghobi Joybari, Behnaz Behzadi, Payam Azadeh, Sam Alahyari

**Affiliations:** 1 *Department of Radiation Oncology, Faculty of Medicine, Shahid Beheshti University of Medical Sciences, Tehran, Iran*; 2 *Faculty of Medicine, Shahid Beheshti University of Medical Sciences, Tehran, Iran*

**Keywords:** Induction chemotherapy, Neoadjuvant chemoradiotherapy, Surgery, Locally advanced rectal cancer

## Abstract

**Background & Objective:**

Currently, neoadjuvant chemoradiotherapy, followed by surgery, is the standard treatment for locally advanced rectal cancer. The use of induction chemotherapy for this tumor is controversial. In this study, the benefits and side effects of induction chemotherapy in locally advanced rectal cancer are evaluated.

**Methods:**

Twenty-nine patients with locally advanced rectal cancer in 2018-2019 were enrolled in this study. Initially, they underwent induction chemotherapy (oxaliplatin 130 mg/m^2^ every 3 weeks and capecitabine 1000 mg/m^2^ twice a day for 14 days every 3 weeks for 2 courses). Then, neoadjuvant chemoradiotherapy (radiotherapy 50.4 Gy/28 for 5 days a week concomitant with weekly oxaliplatin 50 mg/m^2^, as well as capecitabine 825 mg/m^2^/bid on the days of radiotherapy) was administered. After 4 weeks, computed tomography (CT) scan of thorax, pelvis, and abdomen with and without contrast was performed. Total mesorectal surgery was performed 6-8 weeks after the end of radiotherapy. Four courses of adjuvant chemotherapy were applied. Pathologic complete response (pCR), margin, sphincter preservation, and adverse effects were assessed.

**Results:**

In this study, pCR was present in 6 (20.7%) patients. R0 resection was done in 96.05%. Sphincter was preserved in 44.4% of lower rectal tumors. Two patients (6.9%) did not complete adjuvant treatment. Grade 3 adverse effects were documented in 13.7% of cases during induction chemotherapy and 17.2% of cases during neoadjuvant chemoradiation. Mortality was not reported.

**Conclusion:**

Induction chemotherapy, followed by neoadjuvant chemoradiotherapy and surgery, would be an effective and safe modality in locally advanced rectal cancer.

## Introduction

Colorectal cancer (CRC) is currently the third leading cause of cancer death in the world, and its prevalence is increasing in developing countries. CRC is more common in men than in women. The global age-standardized incidence rate of CRC is 19.7 per 100 000 persons, in males is 23.6, and in females is 16.3 (1).

The use of neoadjuvant fluorouracil (FU)-based chemoradiotherapy, followed by complete mesorectal resection and adjuvant chemotherapy, has been shown to result in excellent control of local disease and is, therefore, the standard treatment for advanced rectal cancer. However, the prognosis remains largely unsatisfactory due to a high rate of distant relapse, which is the most common cause of death (2). According to the results of various studies, in the mentioned treatment strategy, patients have little tolerance for chemotherapy after neoadjuvant chemoradiotherapy, and almost 50% of patients cannot receive the planned dose (3-6).

One strategy to improve the side effects of the previous plan is to use induction chemotherapy before neoadjuvant chemoradiotherapy. Induction chemotherapy is associated with a higher tolerance by patients, and it is possible to prescribe a full dose of chemotherapy in this method. Other benefits of this method include shrinkage of locally advanced tumors, which facilitates surgery and early treatment of micrometastases. However, the disadvantages of this method include delays in surgery and lower utility of subsequent radiotherapy (7). 

Today, infusional oxaliplatin and FU/leucovorin (LV) are known as standard treatments for advanced CRC (8, 9). It has been shown that replacing capecitabine with infusional FU/LV is equally effective when used in combination with oxaliplatin (10). Radiotherapy and oxaliplatin with FU and LV (or capecitabine) may result in 15%-28% pathologic complete response (pCR) in locally advanced rectal cancer (11-21). Although in some studies, the addition of oxaliplatin has been associated with increased toxicity during radiation therapy, in some other studies, it has led to an increase in pCR and survival (22, 23). 

Therefore, the aim of this study was to investigate the therapeutic implications of induction chemotherapy followed by neoadjuvant chemoradiotherapy and surgery in locally advanced rectal cancer. We also used oxaliplatin and capecitabine during induction chemotherapy, as well as neoadjuvant chemoradio-therapy, and evaluated its effects and side effects.

## Material and Methods

This study was performed as pilot phase 2, in which 30 new patients with locally advanced rectal cancer were enrolled; who were referred to Imam Hossein hospital in Tehran from 2018 to 2019. Inclusion criteria were age between 18 to 65 years, presence of rectal adenocarcinoma up to a maximum distance of 15 cm from the anal verge, presence of T3 or T4 in terms of TNM, or involvement of lymph nodes (according to the American Joint Committee on Cancer (AJCC) TNM staging system, eighth edition), performance status <2, glomerular filtration rate (GFR) >50, normal liver function (alanine aminotransferase and aspartate aminotransferase less than 2.5 times the maximum normal value), and bilirubin less than 3 times the maximum normal value. 

The study process was completely explained to all patients, and informed consent forms were obtained. Patients with non-adenocarcinoma tumors, T1 N0 or T2 N0 tumors, metastases, previous history of malignancy, history of chemotherapy or pelvic radiotherapy, pregnancy and lactation, recurrent rectal cancer, comorbidity (including myocardial infarction over the past 6 months, congestive heart failure CHF, symptomatic coronary artery disease CAD), and any prohibition to perform chemotherapy or radiotherapy were removed from the study. Routine tests (include Complete blood count, Blood urea nitrogen, Creatinine, Alkaline phosphatase, alanine aminotransferase, aspartate aminotransferase, Bilirubin (Direct, Total), and carcinoembryonic antigen) were done before starting treatment. 

Endoscopic ultrasonography (EUS) or magnetic resonance imaging (MRI) of the abdomen and pelvis with rectal protocol for tumor staging, computed tomography (CT) scans of the thorax, abdomen, and pelvis with and without intravenous (IV)/oral contrast, and endoscopic examination of the entire colon were performed; all patients’ information was recorded. Three patients were staged by an experienced gastroenterologist with EUS and others with MRI. Patients were first treated with induction chemotherapy (oxaliplatin 130 mg/m^2^ every 3 weeks and capecitabine 1000 mg/m^2^/bid for 14 days every 3 weeks for 2 courses) and then neoadjuvant chemoradiotherapy (RT 50.4 Gy/28 fr, oxaliplatin 50 mg/m^2^ weekly, and capecitabine 825 mg /m^2^/bid on radiotherapy days throughout the treatment period). 

Then, 4 weeks later, the patients were examined by CT scans of the thorax, abdomen, and pelvis with and without contrast; 6-8 weeks after the end of radiotherapy, total mesorectal resection was per-formed. Included patients were evaluated for pCR, margin of resection surgery, sphincter preservation, and side effects during treatment. The type of surgery was determined at the time of the initial diagnosis of the tumor. Radiotherapy was performed as a 3-dimensional conformal method with an initial dose of 45 Gy to the whole pelvis and then a 5.4 Gy boost to the tumor with a margin of 2 cm (a total dose of 50.4 Gy). Complications of treatment were assessed according to the Common Terminology Criteria for Adverse Events (CTCAE) version 5 criteria, and dose adjustment was performed based on the British Columbia (BC) Cancer Protocol.

**Statistical Analysis**

Data analysis was performed using SPSS 25 (SPSS Inc., Chicago, Ill., USA). For quantitative variables, mean and SD, and for qualitative variables, absolute and relative frequency were used to present data. Comparison of categorical variables between the study groups was made using the chi-square test or the Fisher’s exact test. P-value<0.05 was considered statistically significant.

## Results

Initially, 30 patients were selected for the study. After chemoradiotherapy for all patients, thoracic, abdominal, and pelvic CT scans were performed with and without contrast to assess metastasis. One patient was excluded from the study due to the presence of pulmonary metastasis after induction chemotherapy. The mean age and mean body mass index (BMI) of the studied patients were 56±9.78 and 25.3±3.1, respectively. Table 1 lists the basic characteristics of the patients. They first underwent induction chemotherapy, then neoadjuvant chemoradiotherapy, and then surgery. Eventually, adjuvant chemotherapy was given to the patients. After surgery, patients were evaluated for pCR, R0 resection, sphincter preservation, and chemotherapy side effects. 

Table 2 shows the clinical and pathological stages of the tumor in patients before and after treatment. Most patients had a tumor with stage T3 (51.7%). In terms of the clinical N stage, 37.9% were N2a patients, and according to the total stage, 48.3% of the participants were IIIC. Among patients, 65.5% (19 patients) had mesorectal fascia involvement. Also, most patients had moderately differentiated tumors. In terms of the total stage, 6 patients (20.7%) had pCR. Further, 44.8% of the patients had a partial response to treatment, and in 34.5% of the participants, the tumor was stable. 

No progressive disease was reported in any of the patients. R1 resection was performed in 1 patient, and R0 resection was performed in others. In terms of tumor distance from the anal verge, lower rectal tumors are located approximately 3-6 cm from the anal verge. Mid rectal tumors are placed from 5-6 cm to 8-10 cm from the anal verge, and upper rectal tumors are placed from 8-10 cm to 12-15 cm from the anal verge. Mid-lower tumors are located in both middle and lower spaces of the rectum, and mid-upper tumors are seen in both middle and upper areas of the rectum. The rate of lower, mid-lower, middle, mid-upper, and upper tumors were 17.2% (6 patients), 13.8% (4 patients), 10.3% (3 patients), 48.3% (14 patients), 10.3% (3 patients) respectively. 

Also, in 23 patients, anal sphincter preservation was possible, which patients with lower and mid-lower tumors had the lowest sphincter preservation rate. In this study, 9 patients had lower and mid-lower tumors, and in 4 patients (44.4%), sphincter preservation was performed. It was also found that there was a significant relationship between the distance from the anal verge and sphincter preservation in patients (*P*=0.041).

**Toxicity**

None of the patients had a treatment interruption. Dose adjustment was performed using BC Cancer Agency guidelines as needed. Fever and neutropenia, leading to hospitalization, were not reported. Complications were divided into three groups as follows: hematological, diarrhea, and neuropathy. In this study, the acute toxicities of grade 3 and above were considered. Overall, 13.7% (4 patients) had side effects from induction chemotherapy, including hematologic (2 patients), diarrhea (1 patient), and neuropathic (1 patient) toxicities. Patients were re-examined for complications after completing the course of chemoradiotherapy. Table 3 shows the number and percentage of toxicities caused by induction chemotherapy and chemoradiotherapy. 

**Table 1:** Patients’ clinicopathologic characteristics

Baseline characteristics	Number	%
Sex		
Male	19	65.5
Female	10	34.5
Performance Status		
0	7	24.1
1	22	75.9
Family History		
Positive	5	17.2
Negative	24	82.8
Smoking		
Positive	6	20.7
Negative	23	79.3
Clinical T stage (pretreatment)		
T2	1	3.4
T3	15	51.7
T4a	5	17.2
T4b	8	27.6
Clinical N stage (posttreatment)		
N1a	4	13.8
N1b	6	20.7
N2a	11	37.9
N2b	7	24.1
N2c	1	3.4
MRF involvement		
Positive	19	65.5
Negative	10	34.5
Tumor grade		
Well differentiated	11	37.9
Moderate differentiated	14	48.3
Poor differentiated	4	13.8

**Table 2:** TNM staging before and after treatment

TNM staging	Before treatment (clinical staging)		After treatment (pathologic staging)	
	Number	%	Number	%
T stage				
pCR	----	----	6	20.7
T1	----	----	2	6.9
T2	1	3.4	6	20.7
T3	15	51.7	13	44.8
T4a	5	17.2	2	6.9
T4b	8	27.6	----	----
N stage				
pCR	----	----	6	20.7
N0	----	----	13	44.8
N1a	4	13.8	4	13.8
N1b	6	20.7	3	10.3
N2a	11	37.9	0	0
N2b	7	24.1	3	10.3
N2c	1	3.4	0	0

**Table 3:** Induction chemotherapy and chemoradiotherapy complications

Complications	Induction chemotherapy		Chemoradiotherapy	
	Number	%	Number	%
Hematologic	2	6.9	2	6.9
Diarrhea	1	3.4	2	6.9
Neuropathy	1	3.4	1	3.4

## Discussion

CRC is the third most common malignancy in men and the second most common malignancy in women, accounting for 8% of cancer-related death worldwide (23). The spread of this cancer is the fastest growing in Asia and Eastern Europe (23, 24). Recent cancer statistics show that in the United States, the incidence of colorectal malignancies is declining due to the timely diagnosis and removal of precancerous lesions by colonoscopy (25). Colorectal malignancies are the third most common cancer among Iranians and occur at younger ages and are on the rise (23, 26). Colorectal malignancies are caused by environmental and genetic factors. 

Today, living standards have risen dramatically across the country; over the past 3 decades, rapid socioeconomic developments have led to a change in lifestyle and a tendency to be sedentary, high-fat and high-protein diets, and reduced consumption of cereals and fiber (28, 29). Proper treatment of rectal cancer can be very important in the prognosis and survival of patients. Choosing the best treatment for advanced local rectal cancer is something that needs further research. Various studies have also been conducted to find predictors of response to neoadjuvant therapy (30). 

Despite the growing tendency to use full neoadjuvant therapy for patients with advanced local rectal cancer, the optimal neoadjuvant treatment for patients remains controversial (31, 32). There is also uncertainty about the effect of adding oxaliplatin to capecitabine in chemoradiotherapy for rectal cancer. Some studies have reported the effect of this drug in increasing the effectiveness of chemotherapy and pCR (33, 34), while some others have indicated an insignificant effect of this drug in chemoradiotherapy (35, 36). Induction chemotherapy before chemoradio-therapy can lead to early treatment of micrometastases. On the other hand, some studies have reported that induced chemotherapy may reduce the effectiveness of chemoradiotherapy due to the development of radiotherapy-resistant cell clones (37). 

This study was performed to investigate the therapeutic implications of induction chemotherapy and then neoadjuvant chemoradiotherapy and surgery in locally advanced rectal cancer. Finally, adjuvant chemotherapy was given to all patients. Of the 30 patients, 1 patient was excluded from the study process after induction chemotherapy due to pulmonary metastasis. Also, 2 patients did not complete the adjuvant treatment, one of which was due to the patient's failure to complete treatment, and the other was due to a grade 4 hematologic complication. 

In this study, the initial end point was pCR. In 20.7% of the patients, pCR was observed. Previous studies have examined the effect of induction chemotherapy before chemoradiotherapy and surgery or as adjuvant therapy, and it was found that induction chemotherapy in Locally Advanced Rectal Cancer (LARC) patients before chemoradiotherapy and surgery reduced grade 3 and 4 toxicity (*P*=0.04) and increased chemotherapy compliance (*P*<0.001) but has no effect on pCR (38, 39). 

Fernandez *et al*. examined the effect of induction chemotherapy before chemoradiotherapy with adjuvant chemotherapy with capecitabine and oxaliplatin in patients with locally advanced rectal cancer. Despite the lack of significant differences in variables, such as pCR and R0 resection, the side effects of adjuvant chemotherapy were significantly higher compared with induction chemotherapy. The pCR in the induction chemotherapy group was 14.3%. Also, the rate of grade 3 and 4 complications in patients receiving induction chemotherapy was 23% (12 patients), which the most common complication was diarrhea (16). The results of this study are similar to our study. Five patients developed grade 3 or higher complications after induction chemotherapy, and the most common complication was hematological complications, seen in 2 patients. 

Chau *et al*. studied the effect of chemotherapy with capecitabine/oxaliplatin before chemoradiotherapy in rectal cancer. In this study, performed on 77 patients, a pCR level of 24% was reported. Also, the rate of complications of grade 3 or higher degrees was 11%. These results were similar to our results (15). In addition, Cercek *et al*. evaluated the effect of FOLFOX chemotherapy before chemoradiotherapy and surgery in 49 patients with rectal cancer. In 27% of the patients, pCR was observed, and 47% had a tumor response of more than 90%. Also, no serious side effects which requires delay in induction chemotherapy or chemoradiotherapy (20). These results were similar to our results were observed. The study also found that there was a significant relationship between tumor distance from the anal verge and preservation of the anal sphincter (*P*=0.041). Also, in patients with lower and mid-lower tumors, there was the lowest rate of sphincter preservation. 

A study of patients with rectal cancer found that keeping the sphincter significantly correlated with multiple factors such as age, BMI, total infiltrated circumference, tumor distance from the anal verge, and depth of invasion. Therefore, it was found that a 5-cm tumor distance from the anal verge is the boundary for the selection of sphincter-preserving resection surgery treatment. Other studies have also shown that tumor distance from the anal verge is the most important factor for considering sphincter-preserving surgery (40, 41). 

Our study has some limitations. Due to the selection of samples from a referral medical center, the generalizability of the results is reduced. If the results are evaluated on the basis of high-risk factors, such as mesorectal fascia involvement, T4 or N2, or lower rectal tumors, it is possible to achieve more accurate results in these patients. In addition, because the research was conducted as a pilot study, the number of samples examined was small. Also, designing more complex studies with a control group to better compare the addition of induction chemotherapy to neoadjuvant chemoradiotherapy with only neoadjuvant chemo-radiotherapy in locally advanced rectal tumors will provide a better understanding of this treatment. The patient follow-up to assess the impact of induction chemotherapy before chemoradiotherapy on patients’ survival may be the subject of future research. 

##  Conclusion

Overall, based on the results of this phase 2 pilot study, it is inferred that induction chemotherapy, followed by neoadjuvant chemoradiotherapy and surgery, is effective in locally advanced rectal cancer and has acceptable toxicity. Therefore, the use of this treatment in these patients is recommended.
